# Gut microbiome and metabolome library construction based on age group using short-read and long-read sequencing techniques in Korean traditional canine species Sapsaree

**DOI:** 10.3389/fmicb.2024.1486566

**Published:** 2024-12-05

**Authors:** Seon-Hui Son, Min-Geun Kang, Anna Kang, Yonggu Kang, Kimoon Kim, Min-Jin Kwak, Minho Song, Younghoon Kim

**Affiliations:** ^1^Department of Agricultural Biotechnology and Research Institute of Agriculture and Life Science, Seoul National University, Seoul, Republic of Korea; ^2^Division of Animal Science and Dairy Science, Chungnam National University, Daejeon, Republic of Korea; ^3^Emerging Pathogens Institute, University of Florida, Gainesville, FL, United States

**Keywords:** microbiome, metabolome, Sapsaree, Illumina sequencing, Nanopore sequencing

## Abstract

This study investigated age-related changes in the gut microbiota and metabolome of Sapsaree dogs through metagenomic and metabolomic analyses. Using Illumina (short-read) and Nanopore (long-read) sequencing technologies, we identified both common and unique bacterial genera in the dogs across different age groups. In metagenomic analysis, Firmicutes were predominant at the family level. At the genus level, *Lactobacillus*, *Streptococcus*, *Romboutsia*, and *Clostridium XI* were the most abundant, and the bacterial genera typically considered beneficial were less prevalent in senior dogs, whereas the genera associated with pathogenicity were more abundant. These findings suggest age-related shifts in gut microbiota composition. Metabolomic analysis showed distinct clustering of metabolites based on the age group, with changes in metabolite profiles correlating with metagenomic findings. Although Illumina and Nanopore methods provided distinctive results, the genera detected by both methods exhibited similar trends across all age groups in Sapsaree dogs. These findings highlight the relationship between ages, metabolite profiles and gut microbiota composition in dogs, suggesting the need for further research to explore this relation in greater depth.

## 1 Introduction

The Sapsaree is a traditional Korean dog (*Canis familiaris*) breed recognized for its long, shaggy coat and drooping ears. In 1992, the Korean government designated the Sapsaree as a “natural monument” to protect it from extinction and preserve its pure bloodline (Gajaweera et al., 2019). Sapsaree has a large body (~60 cm in height), a friendly and gentle nature, and deep loyalty to its host (Cho, 2005). Despite its cultural significance and popularity in Korea, the population of Sapsaree is decreasing owing to limited scientific research on their physiological traits (Kang et al., 2009). Through this study, we aim to investigate the lifestyle and health of Sapsaree by understanding its gut environment via multiomics approaches, including metagenomic and metabolomic analysis.

Gut microbiota refers to the living microorganisms present in a specific environment, in contrast, the microbiome encompasses the complete set of genomes from all the microorganisms in that environment, including not only the microbial community itself but also the structural components, metabolites, and environmental conditions associated with it (Hou et al., 2022). Gut microbiome plays a crucial role in maintaining host health and preventing animal disease (Kwak et al., 2021). Also, recent studies have revealed that the gut microbiota influences various physiological functions, including immune regulation, nutrient absorption, and metabolic processes (Brown et al., 2023). Research specifically on dogs has shown that alterations in the gut microbiota are associated with a range of conditions, such as obesity, gastrointestinal diseases, and even behavioral changes (Kang et al., 2024; Kiełbik and Witkowska-Piłaszewicz, 2024). Understanding the composition and function of the gut microbiota is essential for promoting health and preventing diseases in companion animals (Kwak et al., 2024).

In recent years, next-generation sequencing (NGS) techniques have revolutionized the profiling of microbial communities globally and various studies have been focused on comparison of the efficacy of two types of NGS techniques: the second-generation platform from Illumina (San Diego, CA, United States) and the third-generation platform from Oxford Nanopore Technologies (Oxford, the United Kingdom) (Stevens et al., 2023). MiSeq, a short-read sequencing method using the Illumina platform, provides high accuracy and depth but can be limited in its ability to obtain complete genomic information owing to short-read lengths. By contrast, the Nanopore platform, a long-read sequencing method, can better detect structural variation at the genomic level by providing a longer read length, albeit with a higher error rate (Cha et al., 2023). By comparing these two methods, we aim to evaluate the impact of each sequencing method on analysis of the gut microbiota of Sapsaree.

In this study, we analyze the gut microbiota and gut metabolites of Sapsaree and investigate their correlations to compare the differences between the MiSeq and Nanopore sequencing methods. We also aim to gain a better understanding of the interactions between the gut microbiota and metabolites in Sapsaree to provide valuable foundational information for the health management and disease prevention of companion animals.

## 2 Materials and methods

### 2.1 Animal care

Fecal samples of Sapsaree were obtained from the Korean Sapsaree Foundation (Gyeongsan-si, Korea) to investigate the age-related metagenomic and metabolomic changes. The dogs were categorized into three age groups (*n* = 6 per group): junior, adult, and senior. The junior Sapsaree group consisted of two males and four females aged 1 year. The adult group consisted of five males and one female aged between 4 and 6 years. The senior group consisted of four males and two females aged between 10 and 12 years. The average weights for the junior, adult, and senior groups were 17.6 ± 2.9 kg, 27.7 ± 2.8 kg, and 23.7 ± 2.8 kg, respectively. All dogs were individually housed indoors under controlled conditions. They were fed twice daily, with their daily food intake divided into two meals. Junior Sapsaree dogs were fed with puppy feed for large breeds (Equilibrio, FL, United States), while adult and senior Sapsaree dogs were given Proplan Performance feed from Nestle Purina PetCare Company (MO, United States). Food consumption was monitored immediately after feeding, and the amount of remaining food was recorded. The housing environment was maintained at a constant temperature of 21°C ± 2°C, with a relative humidity of 50% ± 20% and a photoperiod of 12 h (08:00 to 20:00). This study was conducted with approval from the Institutional Animal Care and Use Committee at Chungnam National University (Approval No. 202310A-CNU-179).

### 2.2 Sapsaree fecal sample DNA sequencing using Illumina NextSeq-300

Fecal samples from each group were collected, and genomic DNA was extracted using the DNeasy PowerSoil Pro Kit (Qiagen, Hilden, Germany). The V3–V4 region of the 16S rRNA gene was then amplified using the extracted DNA (V4 amplicon primer set: forward, 515F, 5′-GTGYCAGCMGCCGCGGTAA-3′; reverse, 806R, 5′-GGACTACNVGGGTWTCTAAT-3′). NGS was conducted using the Illumina^®^ NextSeq-300 platform (Illumina, Inc., CA, United States). The quality of the obtained sequences was evaluated using NanoPlot (v1.42.0). The raw V3–V4 amplicon sequencing data have been deposited in NCBI’s SRA with the data accession number SRR30105962.

### 2.3 Metagenomic analysis using Nanopore

DNA samples were quantified using a Qubit 4 fluorometer (Q33226, Invitrogen) and the Qubit dsDNA HS Assay Kit (Q32851, Invitrogen). Amplicon sequencing libraries targeting the V1–V9 regions of the 16S rRNA gene were prepared using the 16S Barcoding Kit 24 V14 (SQK-16S114.24) according to the manufacturer’s instructions. The libraries were sequenced on the MinION Mk1B platform (Oxford Nanopore Technologies, Cambridge, the United Kingdom). The following primers were used: V1 amplicon primer set—forward, 27F (5′-AGAGTTTGATCMTGGCTCAG-3′); reverse, 1492R (5′-GGTTACCTTGTTACGACTT-3′). The quality of the obtained sequences was evaluated using NanoPlot (v1.42.0). The raw V1–V9 amplicon sequencing data have been deposited in NCBI’s SRA with the data accession number SRR30105961.

### 2.4 Metagenomic analysis

**Figure 1:**
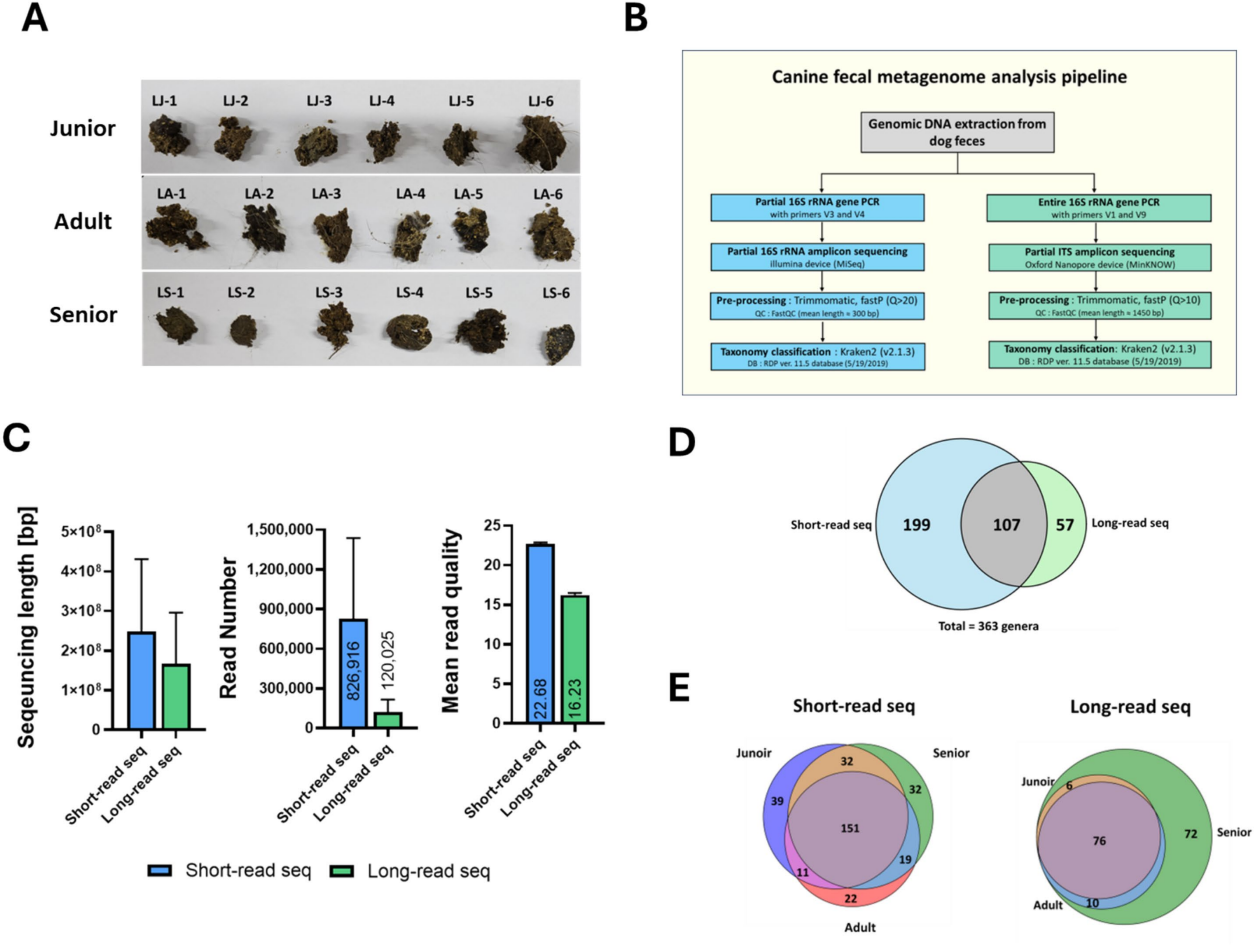
Sampling and metagenomic analysis protocol for Sapsaree. **(A)** Characteristics of fecal samples from Sapsaree dogs. **(B)** Pipeline schematic of metagenomic analysis of fecal samples. **(C)** Comparison between short-read and long-read sequencing. **(D)** Number of the genera analyzed via short- and long-read sequencing techniques. **(E)** Number of genera according to the age group via short- and long-read sequencing techniques. Junior: <1 year old; adult: 4–6 years old; senior: >10 years old.

The amplicon sequences were classified using an analysis pipeline (Figure 1B). Fastq files generated from the Illumina^®^ NextSeq 300 platform were preprocessed using Trimmomatic (v0.39) and fastp (v0.23.4). Paired-end sequences with a read length between 200 and 400 bp and a quality score of at least 20 were classified for 16S rRNA taxonomy using Kraken2 (v2.1.3) with the RDP database (v11.5). Similarly, fastq files generated by the Nanopore MinION Mk1B platform were preprocessed using Porechop (v0.2.4) and fastp (v0.23.4). Single-end sequences with a read length between 1,000 and 2000 bp and a quality score of at least 10 were also classified using Kraken2 (v2.1.3) with the RDP database (v11.5).

For alpha-diversity analysis, the proportions of classified genera were used to calculate the Shannon diversity index (H = −∑(Pi × ln(Pi))) and the Simpson diversity index (∑n_i_ (n_i_ − 1)/(N (N − 1))) to assess microbial diversity in each sample. For bacterial beta-diversity analysis, Bray–Curtis dissimilarity was calculated based on the abundance data. Results were visualized using principal coordinate analysis (PCoA) plots.

### 2.5 Metabolomic analysis

For the extraction of metabolites for GC–MS analysis, each fecal sample was weighed and diluted with ice-cold 100% methanol to achieve a final concentration of 30 mg/mL. The samples were then centrifuged at 15,000 × g for 5 min at 4°C. The resulting supernatant was filtered through a 0.2 μm polyvinylidene fluoride syringe filter. A 0.2 mL aliquot of the filtered supernatant was concentrated using a vacuum concentrator and stored at −80°C until derivatization. The extract was derivatized with 30 μL of 20 mg/mL methoxyamine hydrochloride in pyridine for 90 min at 30°C, followed by 50 μL of *N*,*O*-bis(trimethylsilyl)trifluoroacetamide (BSTFA) for 30 min at 60°C. Fluoranthene was used as an internal standard.

For analysis, a Thermo Trace 1,310 GC system was coupled with a Thermo ISQ LT single quadrupole mass spectrometer (Waltham, MA, United States). Metabolite separation was achieved using a 60 m-long DB-5MS column with an internal diameter of 0.2 mm and a film thickness of 0.25 μm (Agilent, Santa Clara, CA, United States). The sample was injected at 300°C with a split ratio of 1:60 and a helium split flow of 7.5 mL/min. Metabolites were separated using a constant helium flow of 1.5 mL/min and an oven temperature ramp of 5°C/min. The temperature program was started at 50°C (held for 2 min), increased to 180°C (held for 8 min), then increased to 210°C (held for 2.5 min), and finally increased to 325°C (held for 10 min). Mass spectra were acquired over a scan range of 35–650 m/z at an acquisition rate of 5 spectra per second using electron impact ionization. The ion-source temperature was maintained at 270°C. Data analysis, including automated peak detection and metabolite identification, was conducted using the Thermo Xcalibur software by matching mass spectra and retention indices of the samples with those in the NIST Mass Spectral Search Program (version 2.0, Gaithersburg, MD, United States). Metabolite data were normalized based on the intensity of the internal standard, fluoranthene.

### 2.6 Correlation between the metagenomic and metabolomic analyses

The correlation between the microbiota and metabolites was assessed using the integrative analysis pipelines MetaboAnalyst 6.0^1^ and MicrobiomeAnalyst 2.0^2^, with a significance threshold set at *p* < 0.05. Variations in microbial community composition were analyzed at the phylum and genus levels, employing microbiome-metadata correlation analysis. Spearman correlation coefficients were calculated using pairwise correlation analysis to explore the relationships between differential microbiome communities and the presence and concentration of specific metabolites.

### 2.7 Statistics

If not otherwise specified, all values are presented as the mean ± standard deviation and were derived from analysis of samples from six Sapsaree dogs per group. Statistical analysis of the experimental data was performed using the GraphPad Prism 9 software. Nonparametric multiple *t*-tests were conducted to assess statistical significance. Significance levels are indicated by asterisks as follows: * *p* < 0.05.

## 3 Results

### 3.1 Experimental scheme of metagenome study

The metagenomes of 18 Sapsaree dogs, categorized by age (<1 year, 1–6 years, and > 7 years), were analyzed (Figure 1A). The sequencing reads obtained using Illumina MiSeq had an average length of 300.0 bp, while those obtained using Nanopore MinION had an average length of 1405.8 bp, demonstrating a significant difference in the read lengths (Figure 1C). The mean read quality score of the raw data was 22.7 for the short reads obtained by MiSeq and 16.2 for the long reads obtained by MinION, with the short reads exhibiting significantly better quality. When these reads were classified using Kraken2 (v2.1.3) based on the RDP 11.5 database, the short reads were annotated to 306 genera. By contrast, the long reads were annotated to 164 genera (Figure 1D). There were 107 genera in common, i.e., identified by both the sequencing analysis platforms. Despite using the same samples, the genera assignments varied depending on the employed 16S rRNA analysis platform. When examining the number of genera annotated from short reads based on age groups, Illumina-derived short reads identified 233 genera in the junior group, 203 genera in the adult group, and 234 genera in the senior group. Some genera were only identified in specific age groups. However, the age-specific genera were predominantly composed of non-dominant species, with fecal abundance ratios below 0.1% (Figure 1E). When examining the number of genera annotated from long reads based on age group, 164 genera were annotated in the senior group, which included 82 genera from the junior group and 86 genera from the adult group.

### 3.2 Gut microbiome analyzed via short-read sequencing

**Figure 2:**
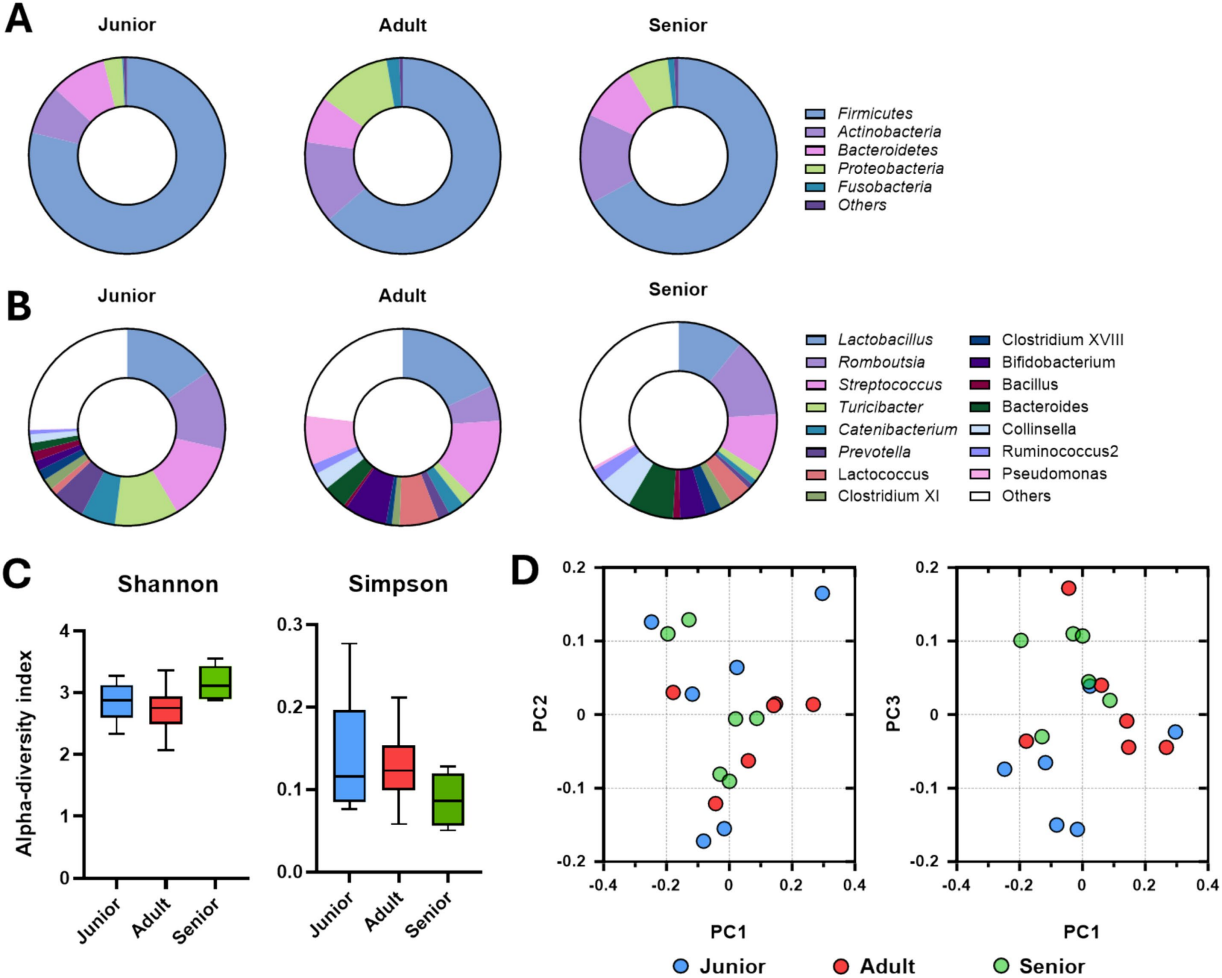
Gut microbiome composition determined via Illumina short-read sequencing. **(A)** Intestinal microbial population at the phylum level according to the age group. **(B)** Intestinal microbial population at the genus level according to the age group. **(C)** Alpha-diversity analysis of gut microbiome samples from Sapsaree dogs (Shannon and Simpson indexes). **(D)** Beta-diversity analysis of gut microbiome samples from Sapsaree dogs. Junior: <1 year old; adult: 4–6 years old; senior: >10 years old.

Composition of the gut microbiome, at the phylum level, of junior, adult, and senior Sapsaree dogs is shown in Figure 2A. The population of the dominant phylum *Firmicutes* gradually decreased with increasing age of Sapsaree dogs, and the populations of *Actinobacteria* and *Proteobacteria* were significantly increased in the adult and senior groups compared to the junior group. The populations of intestinal genera are depicted in Figure 2B; in the senior group, the smallest bacterial populations corresponded to *Lactobacillus* and *Turicibacter* and largest populations were attributed to *Romboutsia* and *Collinsella*. As shown, the Simpson index was numerically decreased compared to CON group (Figure 2C), and the gut microbiome community of the junior group was significantly separated from those of adult and senior groups (Figure 2D).

### 3.3 Gut microbiome analyzed via long-read sequencing

**Figure 3:**
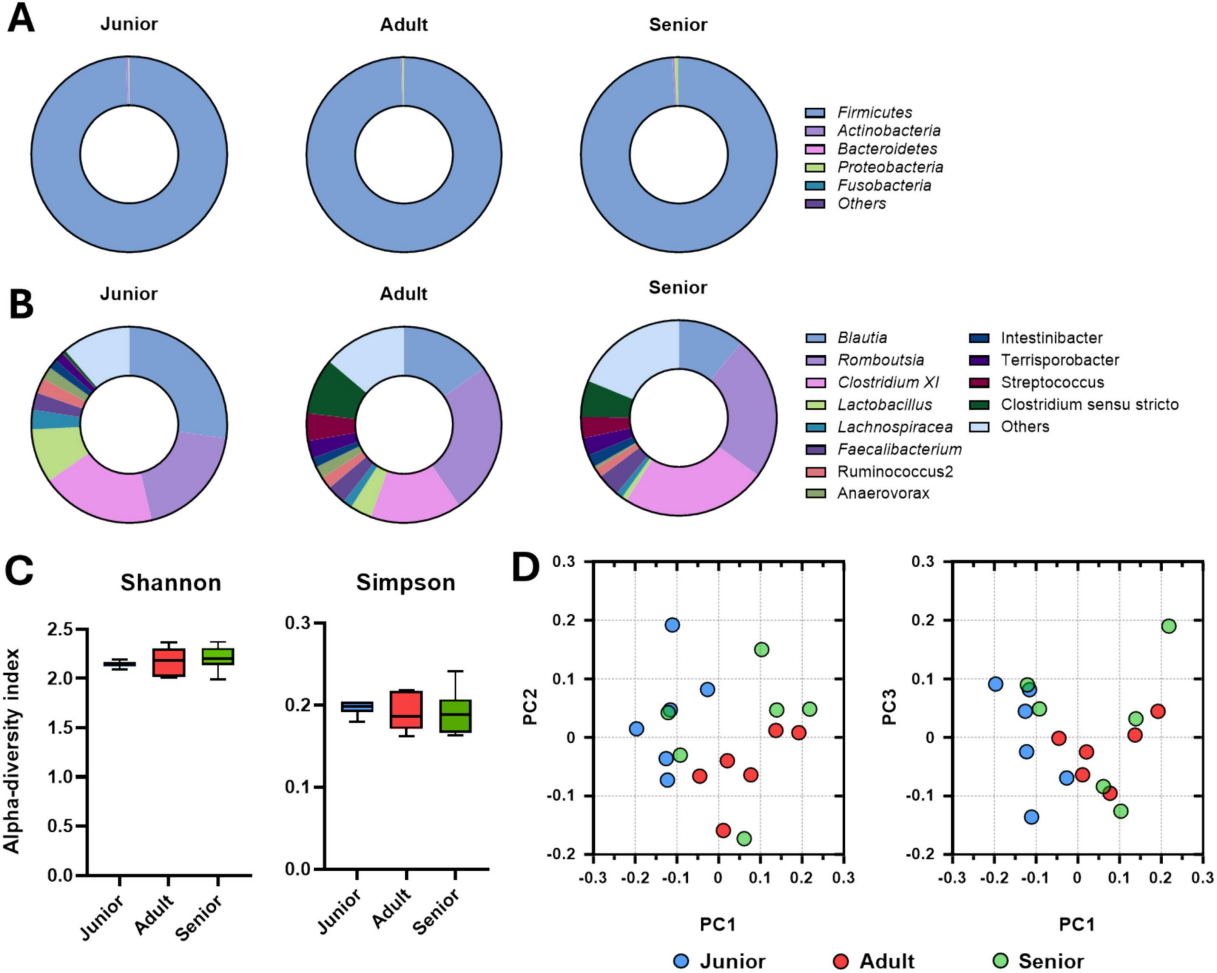
Gut microbiome composition determined via Nanopore long-read sequencing. **(A)** Intestinal microbial population at the phylum level according to the age group. **(B)** Intestinal microbial population at the genus level according to the age group. **(C)** Alpha-diversity analysis of gut microbiome samples from Sapsaree dogs (Shannon and Simpson indexes). **(D)** Beta-diversity analysis of gut microbiome samples from Sapsaree dogs. Junior: <1 year old; adult: 4–6 years old; senior: >10 years old.

Intestinal microbial populations at the phylum and genus levels determined by long-read sequencing are presented in Figures 3A,B. More than 95% of the *Firmicutes* population was found in all age groups. Meanwhile, the populations of *Blautia* and *Lactobacillus* were significantly larger and the populations of *Romboutsia* and *Clostridium XI* were significantly smaller in the junior group compared to the adult and senior groups. There were no differences in alpha-diversity indexes among the groups (Figure 3C); however, the gut microbial community in samples from dogs in the junior group was completely separated from those from dogs in the adult and senior groups (Figure 3D).

### 3.4 Gut metabolome and its predicted metabolism

**Figure 4:**
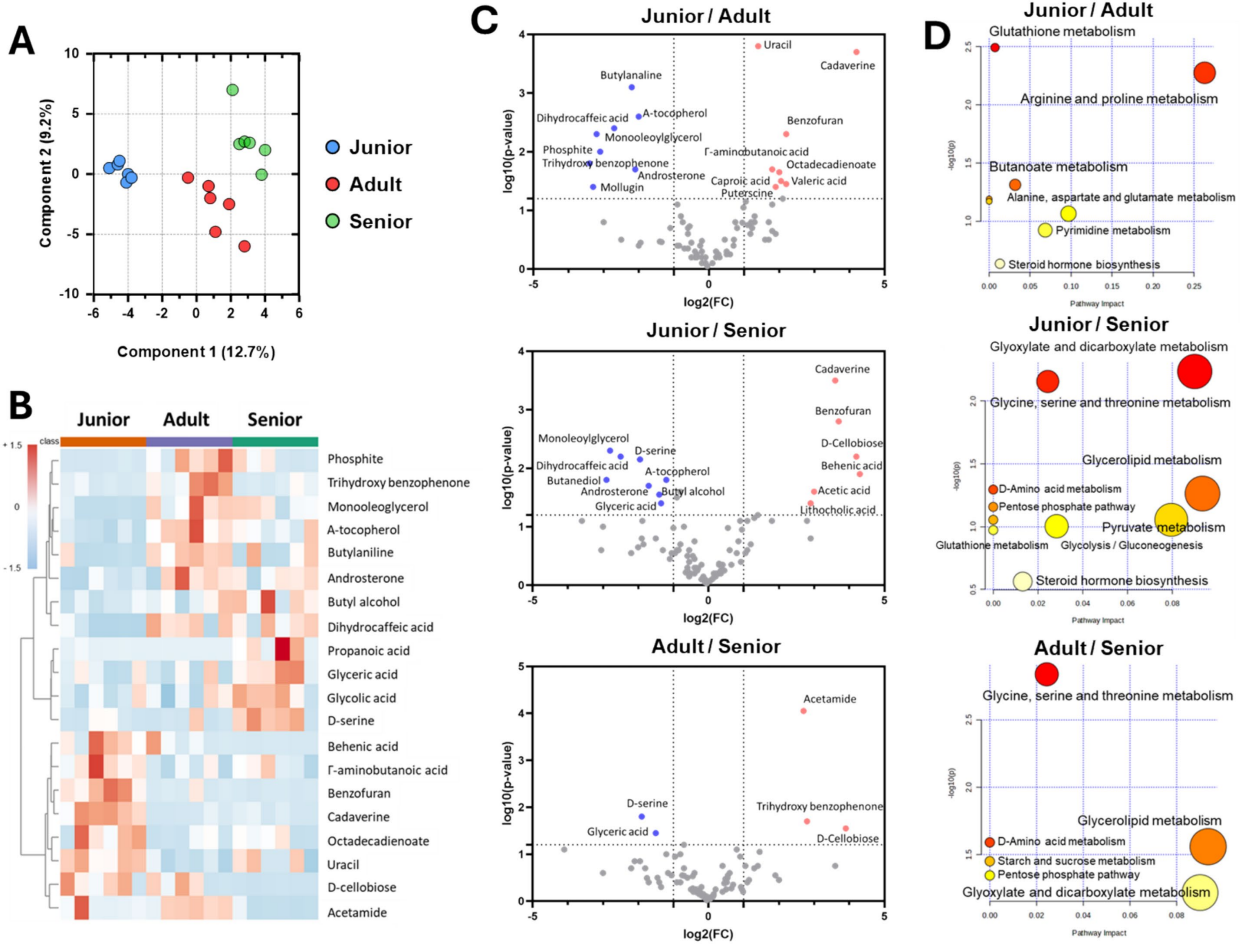
Metabolome analysis of fecal samples from Sapsaree dogs according to the age group. **(A)** PCoA analysis of gut metabolites. **(B)** Representative metabolites in each age group. **(C)** Comparison analysis using volcano plot. **(D)** Predicted metabolism by fecal metabolite abundance. Junior: <1 year old; adult: 4–6 years old; senior: >10 years old.

The metabolome in intestinal contents from Sapsaree dogs was significantly different in dogs of different age groups (Figure 4A). In the junior group, *γ*-amino butanoic acid, benzofuran, cadaverine, octadecadienoate, uracil, and D-cellobiose were the dominant fecal components. Conversely, phosphite, trihydroxy benzophenone, monooleoglycerol, *α*-tocopherol, butylaniline, and androsterone were the dominant components in the adult group, and butyl alcohol, dihydrocaffeic acid, propanoic acid, glyceric acid, glycolic acid, and D-serine were the dominant components in the senior group (Figure 4B). Moreover, comparison analysis using volcano plots demonstrated that the concentrations of cadaverine, uracil, benzofuran, *γ*-amino butanoic acid, and D-cellobiose were significantly higher in the junior group compared to the adult and senior groups and that the concentrations of D-serine, *α*-tocopherol, and glyceric acid were significantly higher in the senior group compared to the other groups (Figure 4C). Based on gut metabolite composition, we predicted higher glutathione, arginine, proline, glyoxylate, dicarboxylate, glycine, serine, and threonine metabolism in the junior group compared to the other groups. Similarly, we predicted that pentose phosphate pathway activity, starch and sucrose metabolism, pyruvate metabolism, and steroid hormone biosynthesis would be significantly increased in the senior group compared to the other groups (Figure 4D).

### 3.5 Relationship between gut microbiome and metabolome

**Figure 5:**
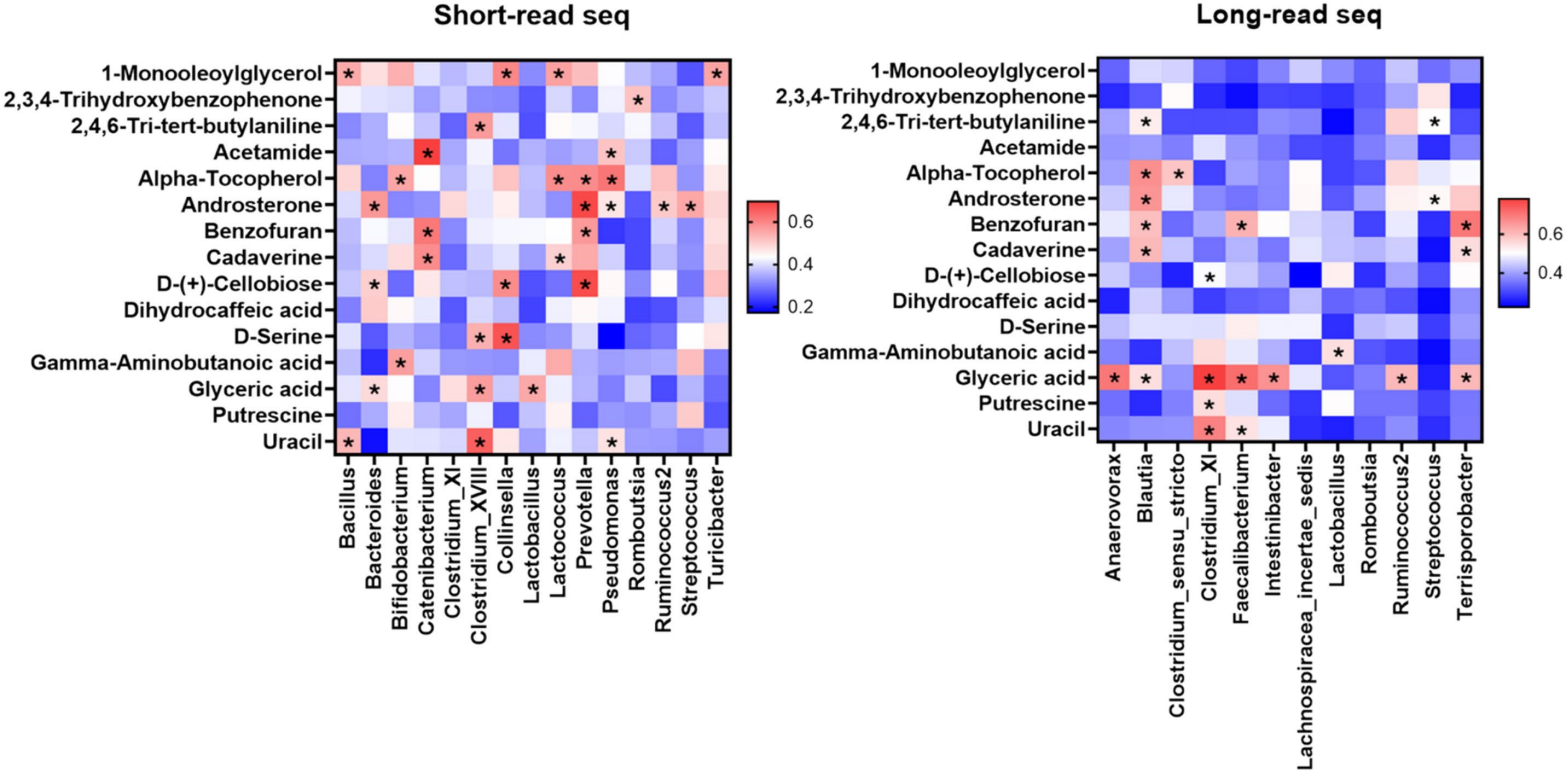
Comparison between gut metagenome (short-read Illumina sequencing and long-read Nanopore sequencing) and metabolome analyses.

Correlation analysis between the gut microbiome and metabolome was performed to investigate the differences between short-read and long-read sequencing. Short-read sequencing results demonstrated that the populations of *Bifidobacterium* and *Lactobacillus* had significant positive correlations with the concentrations of *γ*-amino butanoic acid and *α*-tocopherol and the populations of *Catenibacterium* and *Prevotella* had significant positive corelationships with the concentrations of cadaverine and benzofuran (Figure 5). Additionally, the concentration of D-cellobiose showed a positive correlation with the populations of *Collinsella* and *Prevotella.*

Results from long-read sequencing showed that the concentration of *Blautia* was significantly correlated with the concentrations of 2,4,6-tri-butylaniline, α-tocopherol, androsterone, benzofuran, cadaverine, and glyceric acid (Figure 5). In accordance with the short-read sequencing results, long-read sequencing also demonstrated significant correlation between the concentration of *γ*-amino butanoic acid and the population of *Lactobacillus*. In addition, the concentrations of cadaverine and benzofuran were significantly correlated with the populations of *Faecalibacterium* and *Terrisporobacter*.

## 4 Discussion

Canine fecal metagenome analysis using Illumina and Nanopore technologies exhibited both common and unique genera. At the genus level, genera identified by both the technologies included *Lactobacillus*, *Romboutsia*, *Streptococcus*, *Clostridium XI*, and *Ruminococcus*, with these genera showing similar trends in abundance across different age groups.

Specifically, *Lactobacillus* was observed to have a lower relative abundance in senior dogs compared to junior and adult dogs. *Lactobacillus* is widely recognized as a probiotic, known for its role in strengthening the gut epithelial barrier (Di Luccia et al., 2022; Yao et al., 2024) and exhibiting anti-inflammatory effects that can alleviate gastrointestinal disorders such as inflammatory bowel disease (IBD) (Oh et al., 2018; Aghamohammad et al., 2022). Recent studies have also reported the impacts of *Lactobacillus* on the central nervous system, with its effects on the gut–brain axis being related to γ-aminobutyric acid (GABA) (Patterson et al., 2019; Zhong et al., 2023; Tette et al., 2022). GABA is an important neurotransmitter that primarily functions as an inhibitory agent in the central nervous system (Hampe et al., 2018), reducing anxiety and promoting relaxation (Möhler, 2012). GABA is produced through the decarboxylation of glutamate (Rowley et al., 2012), with a significant proportion of its synthesis occurring via the gut microbiota (Strandwitz, 2018). In this study, we observed a positive correlation with *Lactobacillus* and *Bifidobacterium*, which are known as major GABA producers (Strandwitz et al., 2019), explaining their significant correlation with GABA. The decrease in the proportion of GABA-producing bacteria in senior dogs may be associated with age-related declines in learning and cognitive abilities. Conversely, the heatmap analysis revealed that D-serine levels were higher in senior dogs compared to junior and adult dogs. D-serine acts as a co-agonist for the *N*-methyl-D-aspartate receptor, assisting in receptor activation upon glutamate binding (Ploux et al., 2021). Unlike GABA, D-serine is primarily synthesized within the body rather than by the gut microbiota (Pollegioni and Sacchi, 2010). Increased D-serine levels can be seen as an adaptive response to maintain neurotransmitter homeostasis in case of decreased GABA levels due to aging-related changes in the gut microbiota. However, as no direct correlations between GABA and D-serine synthesis have been reported, additional research is needed to investigate how GABA levels, which are impacted by gut microbiota composition, affect serine levels in the body.

In addition, the relative abundance of *Clostridium XI* was the highest in senior dogs, followed by juniors and adults. *Clostridium XI* is a cluster that includes several pathogenic species (Ohashi and Fujisawa, 2019) such as *Clostridium difficile*, which produces enterotoxins causing colitis (Savidge et al., 2003), and *Clostridium perfringens*, which produces neurotoxins leading to necrotizing enteritis (Cooper and Songer, 2010). However, the *Clostridium* cluster also includes important commensal bacteria that exhibit probiotic effects by producing short-chain fatty acids such as butyrate (Xu et al., 2020). Additionally, species within *Clostridium XI*, such as *Clostridium sordellii* and *Clostridium hiranonis*, are known to secrete 7α-hydroxysteroid dehydrogenases, which deconjugate primary bile acids to form secondary bile acids (Guo et al., 2020). This process not only aids in the circulation of bile acids but also helps inhibit infections caused by *Clostridium difficile*, which is part of the same genus (Buffie et al., 2015). To date, prior research has predominantly focused on the pathogenic aspects of *Clostridium*. Therefore, to gain a comprehensive understanding of the role of *Clostridium* in the gut, further investigation of its potential contributions to intestinal health is needed.

The relative abundance of *Collinsella* was specifically measured using Illumina, unlike with Nanopore. The abundance of *Collinsella* is known to be influenced by the host’s diet; when the host consumes a high-protein diet, the abundance of *Collinsella* decreases, whereas it increases with a fiber-rich diet (Walker et al., 2011; Candela et al., 2016). *Collinsella* showed the lowest abundance in junior dogs, followed by adults and seniors. Junior dogs require more calories for growth and development compared to adult and senior dogs; therefore, puppy food typically has lower carbohydrate content and higher protein and fat content than adult dog food (Jacuńska et al., 2023). This difference in the composition of food for junior and adult dogs can result in junior dogs having the lowest abundance of *Collinsella*. *Collinsella* has a positive correlation with D-(+)-cellobiose, a metabolite not produced by mammalian digestive enzymes but by bacterial enzymes in the gut (Nishimura et al., 2010). This suggests that increased abundance of *Collinsella* due to a high-carbohydrate diet significantly enhances carbohydrate metabolism in the gut, with *Collinsella* performing this necessary function for the host.

Although not detectable with Illumina, the relative abundance of *Blautia* was measured using Nanopore and found to be the highest in senior dogs, followed by adults and juniors. *Blautia* is a genus with members noted as potential probiotics (Mao et al., 2024; Mao et al., 2023). The lipid metabolism capabilities of *Blautia* have also been reported, including the breakdown of glycosylceramides into ceramides, fatty acids, and sphenoid bases for absorption in the gut (Furuya et al., 2010). Notably, mice fed a diet containing 1% purified glycosylceramides as prebiotics showed an increase in gut *Blautia* abundance, accompanied by a significant reduction in blood sugar levels (Hamajima et al., 2016). *Blautia* thus possesses lipid metabolism abilities and has demonstrated positive effects on blood sugar regulation. Additionally, a significant correlation between *Blautia* abundance and obesity has been shown. When mice were given dietary fiber extracted from corn along with a high-fat diet, there was a notable anti-obesity effect characterized by reduced body weight and tissue weight, and the proportion of *Blautia* in the gut microbiome significantly increased (Yang et al., 2016). The diet of the junior Sapsaree dogs in this study included fiber extracted from corn, which is believed to have contributed to the high relative abundance of *Blautia*. *Blautia* abundance is also influenced by the host’s age. An analysis of stool samples from 367 Japanese individuals aged 0–104 years revealed that adults (21–69 years) had higher abundances of *Blautia* and *Bifidobacterium* in their gut microbiome, with decreased *Blautia* abundance observed with increased age and a correlated decline in microbiome diversity (Vaiserman et al., 2017). This finding is consistent with our results showing the lowest *Blautia* abundance in senior dogs.

In contrast to *Blautia*, the highest abundance of *Faecalibacterium* was found in senior dogs, followed by adults and then juniors. *Faecalibacterium* is one of the butyrate producers, and it has been confirmed that the proportion of butyrate producers (including *Faecalibacterium* and *Roseburia*) is reduced in the gut microbiota of patients with IBD compared to those without IBD (Kowalska-Duplaga et al., 2019). In our study, heatmap analysis of the metabolome of junior dogs, which have the lowest abundance of *Faecalibacterium*, revealed a significant increase in the concentration of octadecadienoate (omega-6), a type of unsaturated fatty acid. A previous study found that the abundance of *Faecalibacterium* decreased and that of *Blautia* increased in the gut microbiome of adult men who consumed omega-3 fatty acid supplements (Noriega et al., 2016). This finding is similar to the pattern of gut microbiota composition observed in junior dogs in our study, suggesting an interaction between unsaturated fatty acids, *Blautia*, and *Faecalibacterium* abundance in the gut. However, there is no direct research on the effects of omega-6 fatty acids on gut microbiota changes, indicating a need for further studies on the correlation between omega-6, other unsaturated fatty acids and gut microbiota composition.

This study highlights the differences in metagenome analysis results using Illumina and Nanopore sequencing technologies. Illumina and Nanopore technologies represent short-read and long-read sequencing methods, respectively. Short-read sequencing can provide read sequences up to 600 bp and is cost effective (Pollard et al., 2018; Heather and Chain, 2016). However, there are limitations of short-read sequencing owing to the preferential amplification of repetitive DNA during the random fragmentation and amplification processes. By contrast, long-read sequencing can provide read sequences over 10 kb, recognize repeat sequences, and detect structural variations, although it has lower per-read accuracy compared to short-read sequencing (Adewale, 2020). Due to the shorter sequence length provided by Illumina’s short-read methodology, the results are comparatively easier to annotate, often resulting in an exaggerated detection of microorganism diversity relative to the actual microbial composition (Buetas et al., 2024). Results of this study show that the 16S rRNA metagenome analysis using short reads identified a significantly higher number of genera compared to long reads. The observed differences in gut microbiota analysis between the Illumina and Nanopore methods intuitively demonstrate that each method has its own strengths and limitations, making them complementary to one another. Considering this, it is crucial to interpret data by focusing on genera that are consistently significant for both methods, rather than relying exclusively on a single method. Ultimately, further research comparing short-read and long-read sequencing is necessary.

## 5 Conclusion

In conclusion, this study revealed age-related changes and correlations between the metagenome and metabolome of the gut microbiota in Sapsaree dogs. Metabolome analysis displayed clear clustering tendencies based on age groups, indicating a correlation with the metagenome composition. Metagenomic analysis showed that the genera generally considered beneficial were found in the lowest proportion in seniors, whereas those known as pathogens tended to be higher in proportion, suggesting age-related changes in gut microbiota composition and metabolome. Using Illumina and Nanopore technologies for metagenomic analysis, we observed differences in the results obtained using each technology; however, the genera commonly detected by both technologies showed similar trends in abundance across different age groups. This finding underscores the association between aging and gut microbiota composition, highlighting the need for further research in this area.

## Data Availability

The datasets presented in this study can be found in online repositories. The names of the repository/repositories and accession number(s) can be found at: https://www.ncbi.nlm.nih.gov/, SRR30105962; https://www.ncbi.nlm.nih.gov/, SRR30105961.

## Footnotes

^1^ https://www.metaboanalyst.ca/

^2^ https://www.microbiomeanalyst.ca/
